# A participatory fall-prevention education program (APREAL) for older inpatients in integrated nursing care wards: a quasi-experimental study

**DOI:** 10.1186/s12877-026-08018-0

**Published:** 2026-07-27

**Authors:** Eunja Kim, Yunkyung Kim, Jiwon Park, Eunsu Kim, Jiyoon Park, Hwasook Cho, Gaeun Kim

**Affiliations:** 1 Nursing Department, Keimyung University Dongsan Hospital, Daegu, Republic of Korea; 2 College of Nursing, Keimyung University, Daegu, Republic of Korea

**Keywords:** Accidental falls, Aged, Inpatients, Integrated nursing care service, Self efficacy, Patient education as topic

## Abstract

**Background:**

Falls are a leading cause of preventable harm among hospitalized older adults. In Korean integrated nursing care service (INCS) wards, patients receive nurse-led inpatient care without resident family caregivers; unlike usual wards where family caregivers or privately hired caregivers often remain at the bedside, older patients in INCS wards must independently initiate requests for assistance during daily activities. Evidence on participatory, practice-based fall-prevention education for this setting remains limited.

**Methods:**

A quasi-experimental, nonequivalent control group pretest–posttest design was employed. Seventy patients aged ≥ 65 years in INCS wards of a tertiary hospital were allocated by ward to an experimental (*n* = 35) or comparison group (*n* = 35). The experimental group received APREAL (Active Participatory Real-world Application-based Learning)—intensive education on day 1 followed by daily review and hands-on practice over five days (10–15 min/day)—while the comparison group received routine education. Fall-related knowledge, fall-prevention self-efficacy, and fall-prevention behaviors were measured at baseline and day 5. ANCOVA adjusting for baseline scores was the primary analysis; Bonferroni correction (α = 0.017) was applied. Ceiling effects and ward-level fall-incident rates were examined as supplementary analyses.

**Results:**

ANCOVA revealed that the experimental group had significantly higher adjusted post-intervention scores than the comparison group for all outcomes: fall-related knowledge (F = 27.41, *p* < .001, partial η² = 0.287), fall-prevention self-efficacy (F = 32.85, *p* < .001, partial η² = 0.326), and fall-prevention behaviors (F = 93.14, *p* < .001, partial η² = 0.578). All effects remained significant after Bonferroni correction. Ceiling effects were noted for knowledge and behavior scores in the experimental group. During the study period, the experimental ward had a numerically lower fall rate (0.0 falls per 1,000 patient-days) than the comparison ward (2.3 falls per 1,000 patient-days), though this exploratory comparison was not statistically powered.

**Conclusions:**

APREAL was associated with significant improvements in fall-related knowledge, self-efficacy, and preventive behaviors among older inpatients in INCS wards. The two-cluster design, short follow-up, ceiling effects, and self-report measures warrant cautious interpretation. A multi-center cluster-randomized trial with objective fall-rate outcomes is warranted.

**Trial registration:**

Clinical Research Information Service (CRIS), Republic of Korea, PRE20260625-001; submitted June 25, 2026. Retrospective registration pending.

**Supplementary Information:**

The online version contains supplementary material available at https://doi.org/10.1186/s12877-026-08018-0.

## Background

Falls are among the most common inpatient safety incidents and are widely regarded as a key quality indicator of hospital care [1, 2]. In-hospital falls can cause fractures, head injuries, and soft-tissue damage, potentially leading to prolonged hospitalization, increased healthcare costs, delayed functional recovery, and excess mortality risk—particularly in older adults [2, 3]. The World Health Organization and international expert guidelines consistently emphasize inpatient fall prevention as a priority patient-safety issue [1, 4].

South Korea officially entered a super-aged society in 2025, with adults aged ≥ 65 years exceeding 20% of the population [5]. The proportion of older patients using inpatient services has increased accordingly [6]. Older inpatients face elevated fall risk owing to age-related physiological decline, multimorbidity, polypharmacy, and cognitive impairment [7, 8]. Hospital falls predominantly occur among older patients, with advanced age, prior fall experience, and insufficient fall-prevention education identified as major risk factors [2, 9].

To reduce the caregiving burden on families and improve inpatient care quality, Korea implemented the integrated nursing care service (INCS) system, whereby patients receive comprehensive nursing and caregiving services from hospital-employed nursing staff without resident family caregivers [10]. In contrast to usual Korean wards, where family members or privately hired caregivers may remain at the bedside and assist with toileting, transfers, and ambulation, INCS wards rely primarily on nurses and nursing assistants employed by the hospital. INCS wards have been reported to improve nursing-service satisfaction and revisit intention compared with general wards [11, 12]. However, in the absence of bedside caregivers, older patients may attempt toileting, transfers, or ambulation alone or delay requesting nursing assistance, thereby increasing fall risk [12, 13]. Furthermore, despite enhanced staffing, structural constraints may limit continuous observation, including competing nursing duties, simultaneous call-bell demands, shift handovers, nighttime staffing patterns, and temporary staff absence from the bedside during medication administration or procedures. These constraints make active patient participation in safety behaviors essential [14].

Previous inpatient fall-prevention education has often relied on one-time explanations or printed materials, which may not adequately account for older patients’ cognitive and functional limitations or the ward’s real-world risk contexts, thus limiting sustained learning and behavioral change [15, 16]. International guidance emphasizes patient engagement, repeated education, and practice-based approaches as core strategies for inpatient fall prevention [1, 17, 18]. In Korea, however, evidence on participatory, practice-based fall-prevention education specifically designed for the INCS ward context remains limited. Existing studies have generally applied conventional formats such as video-and-pamphlet sessions, without embedding practice in actual ward environments or incorporating daily reinforcement over consecutive days [15, 19, 20].

Therefore, we developed APREAL (Active Participatory Real-world Application-based Learning), a ward-based, participatory, practice-oriented fall-prevention education program for older inpatients, and evaluated its effects on fall-related knowledge, fall-prevention self-efficacy, and fall-prevention behaviors. As an exploratory secondary objective, we examined fall-incident rates during the study period.

### Hypotheses

Compared with the comparison group receiving routine education, the experimental group receiving APREAL would show greater post-intervention scores in: (1) fall-related knowledge, (2) fall-prevention self-efficacy, and (3) fall-prevention behaviors.

## Methods

### Study design

**Fig. 1:**
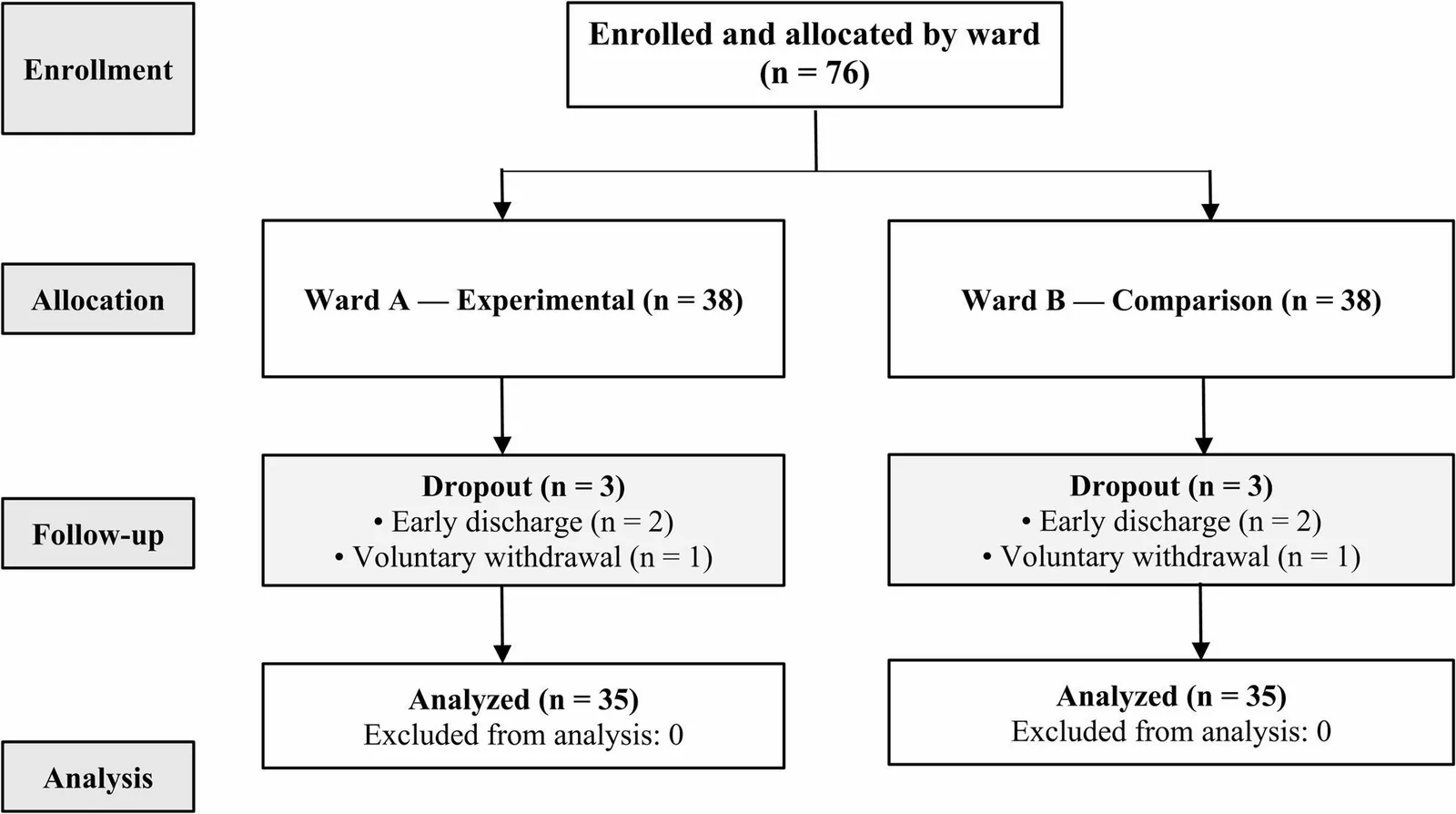
Participant flow diagram. Seventy-six patients who met the eligibility criteria were enrolled in the study and allocated by ward to the experimental group (Ward A, *n* = 38) or the comparison group (Ward B, *n* = 38). During the study period, three participants in each group dropped out. In the experimental group, two participants were discharged early and one withdrew from the study. In the comparison group, two participants were discharged early and one withdrew. Consequently, 35 participants in the experimental group and 35 participants in the comparison group were included in the final analysis

A quasi-experimental, nonequivalent control group pretest–posttest design was used. Reporting followed the Transparent Reporting of Evaluations with Nonrandomized Designs (TREND) statement [21]. A participant flow diagram consistent with the CONSORT extension for non-randomized studies is presented in Fig. 1.

### Setting and participants

The study was conducted in two acute medical INCS wards of a tertiary hospital in a metropolitan city, South Korea, between October 1 and December 12, 2025. Patients from Ward A received the APREAL program (experimental group), while patients from Ward B received routine fall-prevention education (comparison group). The two wards were comparable in bed capacity (45 beds each), nurse-to-patient staffing ratios (approximately 1:7), ward physical layout, and existing fall-prevention protocols. Both wards served acute internal medicine patients and applied identical institutional fall-risk screening procedures (Morse Fall Scale at admission and at each shift change).

Eligible participants were: (1) aged ≥ 65 years; (2) classified as high fall risk (Morse Fall Scale [MFS] ≥ 45); (3) expected length of stay ≥ 5 days; (4) cognitively capable of participating in education and surveys (Mini-Mental State Examination [MMSE] ≥ 18/30); (5) without diagnosed visual or hearing impairment precluding engagement with educational materials; and (6) providing written informed consent. Patients hospitalized due to falls, transferred from the intensive care unit, or experiencing acute confusion or altered consciousness were excluded. Patients hospitalized due to falls were excluded because their admission pathway, injury-related restrictions, and concurrent post-fall management could substantially alter education exposure, mobility status, and baseline motivation compared with the general high-risk inpatient population. Patients transferred from the intensive care unit were excluded because acute clinical instability, severe deconditioning, and rapidly changing mobility restrictions could limit safe participation in repeated hands-on practice and introduce heterogeneity unrelated to the intervention.

The MMSE was used solely as an eligibility screening criterion to confirm adequate cognitive function for participation (score ≥ 18/30); it was not administered as a study outcome measure and MMSE scores were not reported as research results. For this purpose, the Korean version of the MMSE-2 (K-MMSE-2), standardized by Kang, Jang, Kim, and the Korean Dementia Association [22], was employed as part of routine clinical practice at Keimyung University Dongsan Hospital. The K-MMSE-2 was purchased from Inpsyt, Inc., the authorized Korean distributor licensed by PAR, Inc. No MMSE items were reproduced in this manuscript. Permission for the use of this copyrighted instrument was obtained from Inpsyt, Inc. and PAR, Inc., the copyright holder.

Sample size was calculated using G*Power 3.1 based on a prior fall-prevention education study among Korean older inpatients [23], with α = 0.05 (two-tailed), power = 0.80, and effect size d = 0.68 for a two-group independent t-test, yielding a minimum of 35 per group. A 10% attrition allowance indicated a target of approximately 39 participants per group (78 total). Owing to recruitment feasibility within the study period, 76 patients were enrolled (38 per group). After excluding six participants (four early discharges before day 5, two voluntary withdrawals; three per group), 70 participants (35 per group) were included in the final analysis (Fig. 1).

### Intervention: APREAL program

APREAL was developed using the ADDIE (Analysis–Design–Development–Implementation–Evaluation) instructional design model [24], which was selected because it provides a structured and iterative framework for translating needs assessment into educational content, implementation procedures, and evaluation methods. In the Analysis phase, the research team reviewed fall-risk situations in INCS wards and identified patient behaviors requiring reinforcement. In the Design phase, learning objectives were organized around help-seeking, safe mobility, toileting, and post-fall reporting. In the Development phase, animation videos, reminder cards, practice scenarios, and a fidelity checklist were produced. In the Implementation phase, trained research nurses delivered the five-day program. In the Evaluation phase, expert content validity, pilot testing, fidelity monitoring, and pre/post outcome measurement were used to refine and assess the program. Content was derived from a comprehensive analysis of fall-prevention literature, including five intervention studies [15, 18, 20, 25, 26], one clinical guideline [27], and the study hospital’s institutional fall-prevention protocols. Content validity was evaluated by a panel of five experts (two geriatric nursing professors, one fall-prevention specialist nurse, one rehabilitation medicine physician, one patient-safety officer), yielding item-level content validity indices (I-CVI) ranging from 0.80 to 1.00 and a scale-level CVI/Average (S-CVI/Ave) of 0.95 using the proportion-of-relevant-ratings method. A pilot test with three patients was conducted to assess feasibility, comprehension, visual readability, and the appropriateness of the practice sequence; the pilot confirmed that the session length was acceptable and led to refinements in reminder-card font size and video segmentation.

#### Day 1 (intensive education, ~ 15 min)

The first session focused on helping patients understand when, where, and how to request nursing assistance within the INCS context, rather than merely memorizing rules. Animation-based videos (total 7 min 25 s, segmented into short situation-specific clips) depicted real ward situations—bed transfers, corridor ambulation, toileting, and nighttime movement—with repeated modeling of call-bell use and nurse-requesting behaviors. Large-print, illustrated reminder cards covered location- and situation-specific fall risks (bed, corridor, bathroom, nighttime) and corresponding safety actions. The educational paradigm was explicitly reframed from “be careful on your own” to “request nursing assistance and act together.” Reminder cards were placed at the patient’s bedside for continuous reference throughout the hospital stay.

#### Days 2–5 (brief review + hands-on practice, ~10 min/day)

Each session began with a brief review (~ 3 min) using reminder cards and short quizzes, followed by ~ 7 min of supervised hands-on practice in actual ward situations (bed rail operation, safe bed egress, call-bell use, wheelchair transfers, toileting, post-fall response and reporting). Practice involved demonstration by the educator, patient imitation, immediate error correction, verbal encouragement, and repetition. Day 5 included an integrated practice session combining all scenarios and a brief self-assessment. A 12-item implementation fidelity checklist (Additional file 1) was used to standardize delivery; all sessions were delivered by two trained research nurses who received a 4-hour standardized training session prior to the study.

### Comparison condition

The comparison group received routine fall-prevention education per usual ward practice, consisting of a standardized verbal explanation of general fall-prevention precautions and provision of printed educational materials at admission (approximately 5–10 min, one occasion).

### Outcomes and instruments

All outcomes were measured at baseline (hospital day 1, prior to any intervention) and post-intervention (hospital day 5) using the same instruments.

Fall-related knowledge was measured using an 8-item true/false instrument originally developed by Kim and modified by Lee [23, 28], covering intrinsic and extrinsic fall risk factors and prevention strategies (range 0–8; higher = greater knowledge). Kuder–Richardson formula 20 (KR-20) was 0.73 in the original study [28].

Fall-prevention self-efficacy was assessed using the Korean version of the Falls Efficacy Scale (FES) translated and adapted by Choi [29] from Tinetti et al. [30], a 10-item Likert-type instrument measuring confidence in performing daily activities without falling (range 10–100; higher = greater self-efficacy). Cronbach’s α was 0.94 in Choi’s study [29].

Fall-prevention behaviors were measured using a scale developed by Kim and modified by Lee [23, 28], comprising 8 items on a 4-point Likert scale assessing engagement in specific fall-prevention actions (range 8–32; higher = more frequent preventive behaviors). Cronbach’s α was 0.82 in the original study [28].

Exploratory fall-incident data: The number of fall incidents in each ward during the study period (October 1–December 12, 2025) was retrospectively extracted from the hospital’s electronic patient safety incident reporting system. Fall rates were calculated as falls per 1,000 patient-days for each ward.

### Ethical considerations

This study was conducted in full accordance with the ethical principles for medical research involving human subjects set forth in the World Medical Association Declaration of Helsinki (as revised in 2013). The study was approved by the Institutional Review Board of Keimyung University Dongsan Hospital (IRB No. DSMC 2025-09-032). Written informed consent was obtained from all participants. Data were anonymized and stored on a password-protected institutional server. Materials will be retained for three years before destruction per institutional policy.

### Statistical analysis

Analyses were performed using IBM SPSS Statistics version 23.0. Baseline group homogeneity was assessed using χ² or Fisher’s exact test for categorical variables and the independent t-test or Mann–Whitney U test for continuous variables, depending on normality assessed by the Shapiro–Wilk test.

Post-intervention scores were compared between the experimental and comparison groups using analysis of covariance (ANCOVA), with the corresponding baseline score included as a covariate. ANCOVA is recommended for pretest–posttest designs because it adjusts for baseline differences and generally provides greater statistical power than change-score analysis [31]. The assumptions of ANCOVA were assessed prior to analysis, including normality of residuals, homogeneity of variance using Levene’s test, and homogeneity of regression slopes. Effect sizes were reported as partial eta squared (η²p) and interpreted as small (0.01), medium (0.06), and large (0.14) [32].

To ensure consistency with the original analytical plan and facilitate comparison with prior Korean studies, supplementary analyses were conducted using Mann–Whitney U tests on pre-to-post change scores. Hedges’ g was calculated as the effect-size estimate for the change-score comparisons.

Because three primary outcomes were analyzed, a Bonferroni correction was applied to control for type I error, resulting in an adjusted significance level of α = 0.017 (0.05/3). To evaluate potential ceiling effects, the proportion of participants achieving the maximum possible score at posttest was calculated for each outcome. A ceiling effect was defined as occurring when more than 15% of participants achieved the maximum score on the scale [33].

Participants were allocated at the ward level (two clusters). Because only two clusters were included, individual-level analyses could not fully account for potential intracluster correlation, and the intracluster correlation coefficient (ICC) could not be reliably estimated. Therefore, the precision and generalizability of the estimates may be limited, as discussed in the Limitations section.

## Results

### Participant flow

Seventy-six patients who met the eligibility criteria were enrolled and allocated by ward (38 per group). Six dropped out before day 5 (three per group), yielding 70 participants for analysis (Fig. 1). No adverse events related to the intervention were observed.

### Baseline characteristics

Baseline demographic and clinical characteristics were comparable between groups (Table 1). Participants were predominantly aged 70–74 years (35.7%), 54.3% male, and mostly married (81.4%). Fall-risk medication use was equally high in both groups (85.7%). Prior fall-prevention education was reported by 40.0% (experimental) and 48.6% (comparison). Prior fall experience was 20.0% in both groups. No statistically significant between-group differences were found (all *p* > .05). Although not statistically significant, assistive device use was numerically higher in the comparison group than in the experimental group (31.4% vs. 11.4%, *p* = .052).

**Table 1:** Baseline demographic and clinical characteristics of participants

Characteristic	Total (*N* = 70)	Exp. (*n* = 35)	Comp. (*n* = 35)	χ²/t	*p*
Age 65–69	20 (28.6)	10 (28.6)	10 (28.6)	3.72	0.293
Age 70–74	25 (35.7)	15 (42.9)	10 (28.6)		
Age 75–79	19 (27.1)	9 (25.7)	10 (28.6)		
Age ≥ 80	6 (8.6)	1 (2.9)	5 (14.3)		
Sex: male	38 (54.3)	16 (45.7)	22 (62.9)	2.07	0.150
Married	57 (81.4)	28 (80.0)	29 (82.9)	2.84	0.242
Education ≥ middle school	34 (48.6)	18 (51.4)	16 (45.7)	2.59	0.274
Fall-risk medication use	60 (85.7)	30 (85.7)	30 (85.7)	0.00	> 0.999
Prior fall-prevention ed.	31 (44.3)	14 (40.0)	17 (48.6)	0.52	0.470
Prior fall experience	14 (20.0)	7 (20.0)	7 (20.0)	0.00	1.000
Current dizziness	18 (25.7)	12 (34.3)	6 (17.1)	2.62	0.106
Assistive device use	15 (21.4)	4 (11.4)	11 (31.4)	3.89	0.052
Self-rated health: poor	41 (58.6)	19 (54.3)	22 (62.9)	0.53	0.467

Values are *n* (%). Exp. = experimental; Comp. = comparison

### Baseline homogeneity of outcome variables

No significant baseline between-group differences were observed: fall-related knowledge (Z = − 0.911, *p* = .362), fall-prevention self-efficacy (Z = − 0.649, *p* = .516), and fall-prevention behaviors (t = − 1.292, *p* = .201) (Table 2). Internal consistency in the present sample at pretest was KR-20 = 0.68 for fall-related knowledge, Cronbach’s α = 0.92 for fall-prevention self-efficacy, and Cronbach’s α = 0.79 for fall-prevention behaviors.

**Table 2:** Baseline homogeneity of outcome variables

Variable	Exp. M ± SD	Comp. M ± SD	Z / t	*p*
Fall-related knowledge	5.63 ± 2.21	6.06 ± 2.18	−0.911	0.362
Fall-prevention self-efficacy	74.57 ± 25.11	81.03 ± 20.67	−0.649	0.516
Fall-prevention behaviors	22.80 ± 5.42	24.54 ± 5.59	−1.292	0.201

Mann–Whitney U test for knowledge and self-efficacy; independent t-test for behaviors

### Primary analysis: ANCOVA

All assumptions for ANCOVA were met. After adjusting for baseline scores, the experimental group showed significantly higher post-intervention scores than the comparison group across all three outcomes (Table 3). All effects exceeded the Bonferroni-corrected significance threshold (α = 0.017) and were associated with large effect sizes.

**Table 3:** ANCOVA results: adjusted post-intervention scores by group

Outcome	Exp. adj. M (SE)	Comp. adj. M (SE)	F	*p*	Partial η²	95% CI of diff.
Fall-related knowledge	7.80 (0.18)	6.83 (0.18)	27.41	< 0.001*	0.287	[0.95, 2.11]
Fall-prev. self-efficacy	93.95 (2.07)	79.44 (2.07)	32.85	< 0.001*	0.326	[10.99, 22.76]
Fall-prev. behaviors	31.52 (0.53)	23.18 (0.53)	93.14	< 0.001*	0.578	[6.07, 9.24]

Baseline score entered as covariate

*Adj. M* Adjusted mean, *SE* Standard error, *CI* Confidence interval for adjusted mean difference (Exp. – Comp.)

* Significant after Bonferroni correction (α = 0.017)

Specifically, for fall-related knowledge, the adjusted mean difference between the groups was 1.53 (95% CI: 0.95–2.11), indicating a significant group effect, F(1, 67) = 27.41, *p* < .001, partial η² = 0.287. For fall-prevention self-efficacy, the adjusted mean difference was 16.88 (95% CI: 10.99–22.76), with a significant effect of the intervention, F(1, 67) = 32.85, *p* < .001, partial η² = 0.326. Similarly, for fall-prevention behaviors, the adjusted mean difference was 7.65 (95% CI: 6.07–9.24), F(1, 67) = 93.14, *p* < .001, partial η² = 0.578.

### Supplementary analysis: change scores

Change-score comparisons confirmed the ANCOVA findings (Table 4). All three outcomes showed significantly greater pre-to-post improvements in the experimental group (all *p* < .001, all meeting Bonferroni-corrected threshold).

**Table 4:** Supplementary analysis: pre-to-post change-score comparisons

Variable	Group	Pretest	Posttest	Change	Z	*p*	g
Fall-related knowledge	Exp.	5.63 ± 2.21	8.00 ± 0.00	+ 2.37 ± 2.21	−3.98	< 0.001*	1.04
	Comp.	6.06 ± 2.18	6.63 ± 1.68	+ 0.57 ± 0.98			
Fall-prev. self-efficacy	Exp.	74.57 ± 25.11	93.03 ± 11.31	+ 18.46 ± 19.42	−5.68	< 0.001*	1.34
	Comp.	81.03 ± 20.67	80.37 ± 19.11	−0.66 ± 4.83			
Fall-prev. behaviors	Exp.	22.80 ± 5.42	31.29 ± 0.96	+ 8.49 ± 4.93	−6.48	< 0.001*	2.19
	Comp.	24.54 ± 5.59	24.40 ± 5.50	−0.14 ± 2.45			

* *p* < .017 (Bonferroni-corrected). g = Hedges’ g. Values are M ± SD

### Ceiling-effect analysis

A pronounced ceiling effect was observed for fall-related knowledge in the experimental group, where all participants (100%) achieved the maximum score (8/8) at posttest (SD = 0.00). In contrast, 22.9% of participants in the comparison group reached the maximum score.

For fall-prevention behaviors, the experimental group showed a posttest standard deviation of 0.96, with scores ranging from 29 to 32 on a 32-point scale. Notably, 65.7% of participants scored ≥ 31, indicating a substantial ceiling effect. The narrow variance observed at posttest may have inflated the change-score Hedges’ g (2.19), and therefore this effect size should be interpreted with caution.

For fall-prevention self-efficacy, posttest scores ranged from 60 to 100, with 37.1% of participants scoring ≥ 90, suggesting a moderate ceiling effect.

### Exploratory fall-incident analysis

During the 73-day study period, the institutional patient safety reporting system recorded zero fall incidents in Ward A (experimental) and three fall incidents in Ward B (comparison). Based on ward-level patient-days, the fall rate was 0.0 per 1,000 patient-days in Ward A versus 2.3 per 1,000 patient-days in Ward B. However, this comparison should be interpreted with extreme caution: it is not statistically powered (the study was designed for surrogate outcomes, not fall events), the analysis is ward-level rather than participant-level, and unmeasured confounders may account for the difference. The data are presented as descriptive context only and cannot support causal inference regarding APREAL’s effect on fall incidence.

## Discussion

This study developed and evaluated APREAL, a participatory, practice-oriented fall-prevention education program tailored to the INCS ward context. After adjusting for baseline scores via ANCOVA, the experimental group showed significantly higher post-intervention scores for all three outcomes, with large effect sizes (partial η² = 0.287–0.578). These findings suggest that a structured, practice-based educational approach may enhance fall-prevention knowledge, self-efficacy, and self-reported behaviors among older inpatients in INCS wards, extending previous findings from conventional one-time educational interventions [15, 19, 23].

A distinctive feature of APREAL is its focus on actionable safety behaviors feasible without bedside caregivers—requesting nursing assistance via the call bell, refraining from acting alone, and moving slowly—reflecting the unique structural characteristics of INCS wards where patients must independently initiate help-seeking [10, 12, 13]. By repeatedly practicing these behaviors in high-risk ward situations across five consecutive days, the program was designed to facilitate the translation of knowledge into behavioral routines through a demonstration–imitation–feedback–repetition cycle. This approach is consistent with international falls-prevention guidelines that emphasize patient engagement, environmental contextualization, and repeated practice as core educational strategies [1, 17, 18, 26]. In this context, repeated rehearsal of the decision to use the call bell and wait for assistance was intended to help translate general safety information into context-specific help-seeking behavior.

The experimental group achieved a perfect posttest knowledge score (8/8), indicating effective knowledge acquisition but also constituting a pronounced ceiling effect that limits the interpretability of the knowledge outcome. Future studies should employ instruments with greater discriminative capacity at the upper range. Despite this limitation, the combination of multimedia-based initial education and daily reinforcement through reminder cards and quizzes appeared more effective than the single-session verbal-and-pamphlet approach of routine education, which produced only a modest knowledge gain (0.57-point increase). This aligns with evidence that spaced repetition and multimodal delivery are more effective than one-time instruction for older adult learners [15, 18, 19].

The improvement in self-efficacy (partial η² = 0.326) has potential clinical significance, as self-efficacy is a recognized predictor of fall-prevention behaviors and actual fall risk among older adults [30, 34]. The hands-on practice component—in which patients performed and rehearsed safety behaviors in their actual ward environment—may have enhanced mastery experiences, a primary source of self-efficacy in Bandura’s Social Cognitive Theory [35]. However, we did not include a follow-up assessment, so the durability of this self-efficacy gain remains unknown.

The largest ANCOVA effect was observed for fall-prevention behaviors (partial η² = 0.578). However, this finding warrants particular interpretive caution. The experimental group’s posttest SD for behaviors was only 0.96 (compared with a pretest SD of 5.42), indicating extreme score compression near the scale ceiling. This near-zero posttest variance inflates standardized effect sizes (Hedges’ g = 2.19) and may overestimate the true treatment effect. Additionally, the behavior measure relies on self-report, which is susceptible to social-desirability bias, particularly among patients who received intensive, personalized education with repeated interactions with research nurses. Observational or objective behavioral measures in future studies would strengthen this evidence. In addition, the self-reported behavior measure may not fully capture APREAL-specific help-seeking actions, such as call-bell use or refraining from unaided toileting and transfers.

The exploratory fall-incident analysis showed no falls in the experimental ward versus three falls in the comparison ward during the study period. While directionally consistent with the hypothesis, this comparison is severely limited by the absence of statistical power, ward-level confounding, and the short observation window. It is presented as contextual information to complement the surrogate outcomes, not as evidence of fall-rate reduction. A multi-center trial with sufficient sample size and follow-up duration to detect differences in fall rates (e.g., falls per 1,000 patient-days as the primary outcome) is needed to establish clinical effectiveness.

APREAL was operationalized as brief daily sessions (10–15 min) over five days, which enhances feasibility for integration into routine nursing workflows in busy wards. The standardized 12-item fidelity checklist (Additional file 1) and the 4-hour training protocol for research nurses support potential scalability when delivered by ward nursing staff [27]. The program’s inclusion of post-fall response and reporting practice may be particularly relevant in INCS wards, where patients may hesitate to report falls due to embarrassment or fear of blame [13, 14]; embedding reporting practice could contribute to earlier clinical response and more accurate safety surveillance.

Viewed within the broader international literature, a recent scoping review of hospital falls prevention with patient education highlighted limited educational design quality across interventions and called for programs incorporating adult learning principles, age-appropriate materials, and environmental contextualization [17]. APREAL addresses these calls by integrating multimedia instruction, situated learning in real ward environments, scaffolded repetition over multiple days, and immediate feedback—all tailored to older patients’ cognitive and sensory capacities. The INCS context, while specific to Korea, has parallels in other healthcare systems that are transitioning toward nurse-led inpatient care models [10, 11], potentially broadening the applicability of the program’s principles.

### Limitations

Several limitations must be explicitly acknowledged.

First, and most critically, the study used ward-level allocation without randomization, yielding only two clusters (one per arm). Individual-level analyses cannot fully account for intracluster correlation, and the ICC could not be reliably estimated from two clusters. Any unmeasured ward-level confound (e.g., subtle differences in nursing culture, patient case mix, staff attitudes) cannot be disentangled from the intervention effect. This fundamentally limits the internal validity and causal interpretability of the findings. Future studies should employ a multi-center, cluster-randomized design with sufficient clusters to enable mixed-effects modeling or generalized estimating equations (GEE) [36]. Second, outcomes were measured only at hospital day 5, precluding evaluation of long-term behavioral maintenance or actual fall-incidence reduction. It is unknown whether the improvements observed would persist beyond discharge or translate into meaningful reductions in fall rates. Accordingly, the observed day-5 effects should be interpreted as immediate in-hospital changes rather than evidence of sustained behavioral maintenance or longer-term clinical effectiveness. Third, all three outcomes were self-reported and may be susceptible to social-desirability bias and the Hawthorne effect, particularly in the experimental group, which received substantially more attention from research staff than the comparison group. The self-reported behavior measure may also not fully capture APREAL-specific help-seeking actions, such as call-bell use or refraining from unaided toileting and transfers. Fourth, pronounced ceiling effects were observed for knowledge (100% at maximum) and behavior scores (SD = 0.96 at posttest) in the experimental group, likely inflating effect-size estimates and reducing the ability to detect true variance in outcomes. Instruments with broader scoring ranges should be used in future studies. Fifth, the study was conducted in two wards of a single tertiary hospital in one metropolitan city. Generalizability to community hospitals, rural settings, other INCS ward types (e.g., surgical wards), or patients with more severe cognitive impairment (MMSE < 18) is uncertain. Sixth, while Bonferroni correction was applied, testing three outcomes from the same sample carries residual risks of type I error, and the sample size was calculated based on a single outcome.

### Implications for practice and research

Despite these limitations, the findings provide preliminary evidence supporting the feasibility and potential benefit of a ward-embedded, participatory fall-prevention education model for older inpatients in INCS wards. At a practice level, the brief-session format, standardized fidelity checklist, and use of readily available materials facilitate potential integration into routine nursing workflows. At a policy level, the program underscores that sustainable patient-safety education requires not only educational tools but also adequate nursing staffing to deliver repeated education—a consideration aligned with Korean INCS operational guidelines [10] and the WHO World Falls Guidelines [1].

Future research should: (1) employ a multi-center, cluster-randomized controlled trial with fall rates as the primary outcome; (2) include follow-up assessments at 1–3 months post-intervention; (3) use instruments with broader scoring ranges to mitigate ceiling effects; (4) incorporate objective behavioral measures (e.g., nurse-observed call-bell use); (5) explore adaptations for patients with mild-to-moderate cognitive impairment; and (6) include cost-effectiveness analysis to inform policy decisions.

## Conclusions

The APREAL program—a patient-centered, participatory, practice-based fall-prevention education intervention tailored to INCS wards—was associated with significantly higher fall-related knowledge, fall-prevention self-efficacy, and self-reported fall-prevention behaviors among older inpatients compared with routine education, with large effect sizes on ANCOVA. The exploratory fall-incident data were directionally favorable but not statistically powered. The two-cluster design, short follow-up, self-reported outcomes, and observed ceiling effects limit the strength of causal inferences. Nevertheless, the program’s brief-session format, ward-embedded design, and standardized delivery suggest feasibility for integration into routine practice. A multi-center cluster-randomized trial with objective fall outcomes is needed to confirm effectiveness and inform wider implementation.

## Supplementary Information

Additional file 1: APREAL 12-item implementation fidelity checklist.

## Data Availability

The data presented in this study are available on reasonable request from the corresponding author (G.K.; eun0325@kmu.ac.kr). The data are not publicly available due to participant privacy considerations and institutional data governance policies.

## Abbreviations

APREAL: Active Participatory Real-world Application-based Learning
INCS: Integrated Nursing Care Service
MMSE: Mini-Mental State Examination
K-MMSE-2: Korean Mini-Mental State Examination, 2nd Edition
MFS: Morse Fall Scale
ANCOVA: Analysis of Covariance
FES: Falls Efficacy Scale
IRB: Institutional Review Board
ADDIE: Analysis–Design–Development–Implementation–Evaluation
I-CVI: Item-level Content Validity Index
S-CVI/Ave: Scale-level Content Validity Index/Average
KR-20: Kuder–Richardson Formula 20
ICC: Intracluster Correlation Coefficient
TREND: Transparent Reporting of Evaluations with Nonrandomized Designs
CI: Confidence Interval
SD: Standard Deviation
SE: Standard Error
