# Supplementary material for: A participatory fall-prevention education program (APREAL) for older inpatients in integrated nursing care wards: a quasi-experimental study
Source: BMC Geriatr. 2026 Jul 27;26:1333. doi: 10.1186/s12877-026-08018-0 (PMC13693018; doi:10.1186/s12877-026-08018-0)
Supplement: Supplementary file 1 [file 12877_2026_8018_MOESM1_ESM.docx]

**Additional file 1**

**APREAL 12-Item Implementation Fidelity Checklist**

Study: *A participatory fall-prevention education program (APREAL) for older inpatients in integrated nursing care wards: a quasi-experimental study*

**Instructions:** For each participant, the research nurse completes this checklist after every session. Check (✓) if the item was delivered as intended, or mark (✗) if not delivered and note the reason. All 12 items should be completed over the 5-day program.

Participant ID: ____________	Ward: ____________	Nurse: ____________	Date: ____________

| **No.** | **Checklist Item** | **Implementation Detail** | **Done (✓/✗)** |
| --- | --- | --- | --- |
| **Day 1: Intensive Education Session (~15 min)** | | | |
| 1 | Introduced self and explained purpose of fall-prevention education within INCS ward context | *Reframed paradigm: “request nursing assistance and act together” rather than “be careful on your own”* | □ |
| 2 | Played animation-based fall-prevention video (total 7 min 25 s, segmented clips) | *Situation-specific clips: bed transfers, corridor ambulation, toileting, nighttime movement; modeling of call-bell use and nurse-requesting behaviors* | □ |
| 3 | Distributed large-print illustrated reminder cards and reviewed each scenario | *Cards covered: bed, corridor, bathroom, nighttime; location-specific fall risks and corresponding safety actions* | □ |
| 4 | Placed reminder cards at patient’s bedside for continuous reference | *Verified patient can see and reach cards; confirmed understanding of key messages* | □ |
| **Days 2–5: Brief Review + Hands-on Practice (~10 min/day)** | | | |
| 5 | Conducted brief review (~3 min) using reminder cards and short quizzes | *Reviewed previous day’s content; corrected any misconceptions* | □ |
| 6 | Demonstrated correct bed rail operation and safe bed egress technique | *Patient imitation → immediate error correction → verbal encouragement → repetition* | □ |
| 7 | Practiced call-bell use in simulated situations (bed, bathroom, nighttime) | *Ensured patient can locate, reach, and activate call bell from multiple positions* | □ |
| 8 | Practiced safe wheelchair transfer technique | *Lock brakes → position footrests → stand with support → turn and sit; request nurse assistance* | □ |
| 9 | Practiced safe toileting procedure (bathroom transfer and use) | *Grab bar use, non-slip footwear check, call-bell use before and after toileting* | □ |
| 10 | Practiced post-fall response and reporting procedure | *Remain still → call for help → do not attempt to stand alone → report to nursing staff; addressed barriers to reporting (embarrassment, fear of blame)* | □ |
| **Day 5: Integration and Self-Assessment** | | | |
| 11 | Conducted integrated practice session combining all scenarios | *Patient performed full sequence: bed egress → call bell → corridor → bathroom → wheelchair → post-fall response* | □ |
| 12 | Completed brief self-assessment of confidence in fall-prevention behaviors | *Patient verbalized key safety actions; educator confirmed understanding and provided final encouragement* | □ |

**Notes / Reasons for non-delivery:**

_______________________________________________________________________________

_______________________________________________________________________________

_______________________________________________________________________________

**Fidelity criterion:** A session was considered delivered with fidelity if all applicable items for that session were checked (✓). The program was considered delivered with fidelity for a participant if all 12 items were completed across the 5-day period.

*APREAL = Active Participatory Real-world Application-based Learning; INCS = Integrated Nursing Care Service.*
